# Population-specific demography and invasion potential in medfly

**DOI:** 10.1002/ece3.33

**Published:** 2011-12

**Authors:** Alexandros D Diamantidis, James R Carey, Christos T Nakas, Nikos T Papadopoulos

**Affiliations:** 1 Department of Agriculture, Crop Production and Rural Environment, University of ThessalyPhytokou St. N, Ionia 384 46, Magnisias, Greece; 2 Department of Entomology, University of CaliforniaDavis, California 95616

**Keywords:** Biological invasions, *Ceratitis capitata*, Intrinsic rate of increase, Life history traits, Population growth rates

## Abstract

Biological invasions are constantly gaining recognition as a significant component of global change. The Mediterranean fruit fly (medfly) constitutes an ideal model species for the study of biological invasions due to its (1) almost cosmopolitan geographic distribution, (2) huge economic importance, and (3) well-documented invasion history. Under a common garden experimental set up, we tested the hypothesis that medfly populations obtained from six global regions [Africa (Kenya), Pacific (Hawaii), Central America (Guatemala), South America (Brazil), Extra–Mediterranean (Portugal), and Mediterranean (Greece)] have diverged in important immature life-history traits such as preadult survival and developmental times. We also tested the hypothesis that medfly populations from the above regions exhibit different population growth rates. For this purpose, data on the life history of immatures were combined with adult survival and reproduction data derived from an earlier study in order to calculate population parameters for the above six populations. Our results clearly show that medfly populations worldwide exhibit significant differences in preadult survival, developmental rates of immatures and important population parameters such as the intrinsic rate of increase. Therefore, geographically isolated medfly populations may share different invasion potential, since population growth rates could influence basic population processes that operate mostly during the last two stages of an invasion event, such as establishment and spread. Our findings provide valuable information for designing population suppression measures and managing invasiveness of medfly populations worldwide.

## Introduction

Biological invasions represent one of the major components of global change (Vitousek et al. 1996). Globalization in many aspects of human activity, such as international trade, transport, and travel during the latter half of the 20th century, has resulted in an increasing number of nonindigenous species breaching their natural dispersal barriers and becoming established in new regions (Perrings et al. 2005). This spread of exotic species in previously unoccupied areas poses a major threat to global biodiversity, ecosystem structure and integrity, public health and economy (Lodge 1993; Mooney and Cleland 2001; Pimentel et al. 2001, 2005). As the frequency of biological invasions is constantly increasing, so does the number of studies attempting to identify biological traits of potential invaders (Kolar and Lodge 2001), features of ecosystems susceptible to invasion (Levine 2000; France and Duffy 2006), and finally the role of biogeographic, economic, and demographic factors on the level of invasion by alien species (Pysek et al. 2010). Furthermore, the economic, ecologic, and social impacts of biological invasions have launched strict legislative efforts to prevent the introduction of invasive alien species (Hulme et al. 2009), and large-scale eradication projects when prevention is impossible (Myers et al. 2000; Simberloff 2009).

Despite their negative effects, biological invasions offer an excellent opportunity for evolutionary studies. Invading populations of alien species are often subjected to different selective regimes than those in their native range. Under new abiotic and biotic conditions newcomers must survive, breed successfully, compete with other native species of the recipient community, avoid natural enemies and predators, and finally promote further survival and reproduction by expanding their range to suitable habitats (Carroll and Dingle 1996). Evolutionary response to these environmental challenges often leads to new life-history optima of the invading population (Yoshida et al. 2007). Therefore, introduced populations of an invasive species may ultimately diverge in important life-history traits as a response to selection pressures of the novel environment. Divergence of a specific life-history trait is a complicated biological process depending on the frequency and the magnitude of invasions events, invasion history, the force and direction of natural selection, and the genetic architecture of the invading population (Diamantidis et al. 2008a).

**Figure 1:**
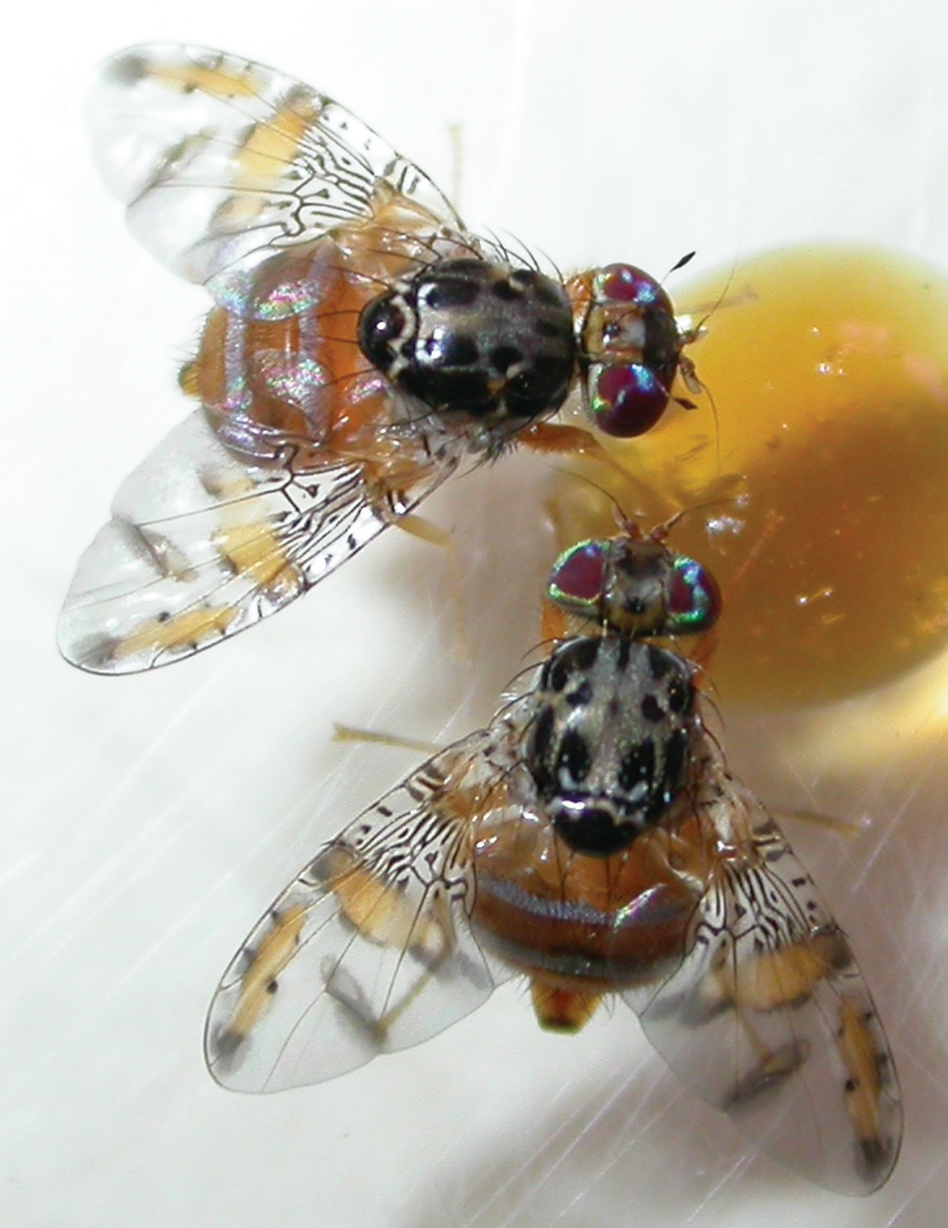
Male and female Mediterranean fruit flies.

The Mediterranean fruit fly (medfly), *Ceratitis capitata* (Wiedeman) (Fig. 1) holds an impressive record of successful invasions promoted by the growth and development of international fruit trade. Medfly is also considered as one of the most destructive pests in global production of fresh fruit and vegetables with a host range that exceeds 300 plant species (Liquido et al. 1991). *C. capitata*, as other frugivorous tephritids, possesses a typical set of morphological, physiological, and demographic traits that contribute to its great invasiveness (Yuval and Hendrichs 2000). Larval development takes place within the sheltered environment of host fruit, minimizing thus mortality risk from predators and parasites. Furthermore, medfly larvae have the ability to recognize, migrate to, and exploit fruit parts of supreme nutritional value (Zucoloto 1987, 1991). Once their development is complete, medfly larvae abandon host fruit in order to pupate in small depths within soil, following a specific daily rhythm that again reduces predation risk (Yuval and Hendrichs 2000). Medflies devote a significant amount of their life span seeking and feeding on highly nutritional substrates, containing protein and carbohydrates, which contribute to a high reproductive output. Finally, by analyzing more than 1000 individual reproductive patterns, Novoseltsev et al. (2004) concluded that female fecundity during the maturity stage in medfly follows a constant rate of egg laying.

Taking into consideration these features that ensure biological success and adaptation in ecologically diverse habitats, it is not surprising that medfly is considered as a global invader that today exhibits an almost cosmopolitan geographic distribution. The genetic structure and affinity among medfly populations worldwide has been investigated in a series of detailed studies conducted during the past 20 years (Malacrida et al. 1998; Bonizzoni et al. 2000; Gasperi et al. 2002; Malacrida et al. 2007). Despite the fact that the above studies lack concrete evidence for the African continent as a whole, they provide evidence that medfly originates from the sub-Saharan East region of Africa (Kenya). From its area of origin in central east Africa, medfly continued the route of its global invasion by establishing populations progressively to the Iberian Peninsula and then to other coastal and eastern Mediterranean regions (Gasperi et al. 2002). Medfly invasion to Australia represents probably a secondary colonization event from Mediterranean regions (Bonizzoni et al. 2004), whereas the establishment in many regions of the New World is mainly attributed to the growth and development in global fruit trade and increased human mobility (Malacrida et al. 2007).

It is clear that besides its great contribution to aging and demographic research (Carey 2003), medfly constitutes an ideal model species for the study of biological invasions due to its (1) almost cosmopolitan geographic distribution, (2) huge economic importance, and (3) well documented, by numerous historical records, invasion history outside the African core range (De Breme 1842; Fimiani 1989; Carey 1996; Myers et al. 2000). Medfly life history has been thoroughly investigated during the past few decades mainly within the frameworks of population biology and pest management (Diamantidis et al. 2008, and references therein). Earlier studies, utilizing mostly laboratory-adapted medfly populations, and few wild ones from a limited number of geographic localities, generally assume a rather similar demographic profile. However, in a series of previous studies we demonstrated that medfly populations from different global regions have diverged in (1) important adult life-history traits (Diamantidis et al. 2009), (2) sexual signaling patterns (Diamantidis et al. 2008b), and (3) demographic aspects of domestication to captivity (Diamantidis et al. 2011). Here we test the hypothesis that, apart from adult survival and reproductive patterns, medfly populations from six global regions have also diverged in vital life-history traits of immatures, such as preadult survival and immature developmental period. We also test the hypothesis that medfly populations from the above regions exhibit different population growth rates. This fact may affect the invasion potential of different medfly populations, since population growth rates may influence basic population processes that operate during invasion events (Liebhold and Tobin 2008). For this purpose, data on the life history of immatures are combined with adult survival and reproduction data derived from an earlier study (Diamantidis et al. 2009) in order to calculate population parameters for the above six populations as a mean of gaining insights on the invasive potential of this important pest.

## Materials and Methods

### Experimental conditions and flies used

The experiments were conducted in the laboratory of Entomology and Agricultural Zoology at the University of Thessaly during summer 2005–autumn 2008, at 25 ± 1°C, 65 ± 5% R.H., and a photoperiod of L14:D10 with photophase starting at 0700 h. Light was provided by daylight fluorescent tubes and by natural light from four windows with the intensity inside the test cages ranging from 1500 to 2000 Lux.

We tested six medfly populations originating from the following global regions: (1) Extra–Mediterranean (Portugal, Madeira, lat: 32.74, lon: -16.98, host: *Prunus persica*), (2) Africa (Kenya, Nairobi, lat: -1.27, lon: 36.8, host: *Coffea arabica*), (3) Mediterranean (Greece, Chios, lat: 38.47, lon: 25.99, host: *Citrus aurantium*), (4) Central America (Guatemala, Antigua, lat: 14.56, lon: -90.74, host: *C. arabica*), (5) Pacific (Hawaii, Kauai, lat: 22.03, lon: -159.32, host: *C. arabica*), and (6) South America (Brazil, Petrolina, lat: -9.40, lon: -40,49, host: *Psidium guajava*). Details on the climatic conditions prevailing in the above regions are given in Diamantidis et al. (2009). Collections of wild medflies from the above fruit hosts were made by harvesting infested fruits and allowing the larvae to pupate under laboratory conditions. Pupae retrieved were transported by a courier agency to our laboratory in Volos, Greece. A total of 500–100 pupae were introduced for each medfly population. Since several medfly traits may be affected by host fruit species (Krainacker et al. 1987), we reared all six populations for one generation under identical laboratory conditions and used the F_1_ generation for the comparison among medfly populations. Rearing of wild flies was accomplished by keeping adults, after emergence, in groups of about 100 individuals in wooden, (30 ´ 30 ´ 30 cm), wire-screened cages provided with water and a standard adult diet consisting of a mixture of yeast hydrolysate, sugar, and water (YS) at 4:1:5 ratio, respectively. Females were allowed to oviposit on 5-cm-diameter hollow, plastic hemispheres of red color (domes) that were artificially punctured with 40–50 evenly distributed holes on their surface. Eggs were deposited on the inner surface of the dome. Each dome was fitted in a 5-cm-diameter hole made on the cover of a 5.5-cm-diameter plastic petri dish. Water was placed in the base of the petri dish in order to maintain humidity levels beneath the dome adequate enough for female oviposition (Boller 1985). A plastic cup containing 0.5 mL of orange juice was also placed in the base of the petri dish to stimulate oviposition.

On a previous study, under a common garden experimental approach, we demonstrated that the above populations have evolved different survival and reproductive schedules (Diamantidis et al. 2009). Female cohorts were classified into either short lived (Guatemala, Hawaii, and Kenya) or long lived (Portugal, Brazil, and Greece). Males on the other hand were grouped differently with only the Guatemalan population being short lived and the remaining five forming a group of long-lived populations. Although lifetime fecundity rates were similar among populations, large differences were observed in their age-specific reproductive patterns.

### Immature traits

Immatures of each medfly population were reared individually (3 mL of food per individual) on an artificial diet (Boller 1985) in order to avoid density-dependent effects that occur under crowded conditions. Eggs were collected with artificial oviposition substrates (domes) offered to the six medfly populations for a 2-h period (0700–0900 h). For all six populations used 200–225 eggs were collected. Eggs were observed at 2-h intervals under a binocular stereoscope (Leica MZ 12, Leica Microsystems, Wetzlar, Germany. Objectives: PLAN APO 1.0X, Eyepieces: 10x/21B, Zoom Factor: 0.8-10x) to determine hatching percentage and duration of embryonic development for all populations. Developing larvae and pupae were observed three times a day to determine the duration of larval and pupal stage, respectively. Overall, the following data were collected: (1) stage-specific survival (%) for all stages of immature development, and duration of (2) embryonic (egg), (3) larval, (4) pupal, and (5) total immature (egg-adult) development.

### Population increase parameters

Adults obtained from the above immatures were used to determine the survival and fecundity patterns of the above six medfly populations (Diamantidis et al. 2009). These data (not given here) were combined with those on developmental times and survival rates of immature stages to compute population parameters following standard methods (Carey 1982, 1984, 1993; Papadopoulos et al. 2002).

### Statistical analyses

Since normality tests failed for all duration of immature stages variables (Kolmogorov–Smirnov test used), survival analysis methodology was followed for all time to event data. Kaplan–Meier estimators of embryonic (egg), larval, pupal, and egg to adult developmental periods were calculated for each population. Pairwise comparisons of the respective periods between populations were conducted using the log-rank (Mantel–Cox) test. Effects of population on the duration of immature stages were assessed using the Cox proportional hazards model, λ(*t*) = λ_0_(*t*) e^ηi (**X**i)^ (1) (Collett 2003). The estimated Cox model with population as covariate is given by (1) where η_i_(**X**_i_) = β_1_population1_i_+β_2_population2_i_+β_3_population3_i_+β_4_population4_i_+β_5_population5_i_ (1a) is the linear component of the model, λ(*t*) denotes the probability of an individual reaching the next stage at any given time conditional on the observed covariates and λ_0_(*t*) the respective probability for the individual with baseline characteristics. All covariates are indicator functions with population1_i_, population2_i_, population3_i,_ population4_i_, and population5_i_ = 1 when subject originated from Guatemala, Greece, Brazil, Portugal, and Hawaii, respectively (else 0), so that Kenya forms the baseline. The covariate effects on egg, larval, pupal, and egg to adult developmental periods are measured by the beta coefficients in the linear component of the model. A c^2^ test was conducted to analyze the survival rates of immature stages with the application of the Bonferoni correction for multiple per two comparisons (Sokal and Rohlf 1995). Confidence intervals for population parameters were estimated based on the 2.5 and 97.5 percentiles of a bootstrap distribution resampled 1000 times (Davison and Hinkley 1997). Data analyses were performed using the SPSS 17.0 (SPSS Inc., Chicago, IL, USA) and the R 2.10 version (http://www.r-project.org).

## Results

### Immature traits

Significant differences in immature survival were observed among the six populations tested for all stages apart from the pupal one (Table 1). Egg to adult survival ranged between 67.5% (Portugal) and 82.5% (Greece). The lowest mortality was observed during the pupal stage for all populations except for flies originating from Greece (Table 1). Embryonic and larval survival was greater than 79% for all populations (Table 1).

**Table 1:** Survival of immature stages of six medfly populations reared on an artificial diet in the laboratory (25°C)

Population	Survival (%)			
	
	Egg	Larvae	Pupae	Egg–Adult
Kenya	82.3 b	87.8 ab	96.0 a	69.5 b
	(*n* = 210)	(*n* = 173)	(*n* = 152)	
Portugal	84.0 b	84.7 b	94.8 a	67.5 b
	(*n* = 219)	(*n* = 184)	(*n* = 156)	
Greece	96.5 a	90.6 ab	94.2 a	82.5 a
	(*n* = 200)	(*n* = 193)	(*n* = 175)	
Hawaii	89.0 ab	86.5 b	99.3 a	76.5 a
	(*n* = 200)	(*n* = 178)	(*n* = 154)	
Brazil	79.3 b	96.0 a	95.8 a	72.9 a
	(*n* = 222)	(*n* = 176)	(*n* = 169)	
Guatemala	95.0 a	86.5 b	98.8 a	81.2 a
	(*n* = 202)	(*n* = 192)	(*n* = 166)	
c^2^	46.8274	14.8972	10.3614	12.3906
df	5	5	5	5
*P*	<0.001	0.01	0.06	0.02

Percentages within the same column followed by the same letter are not statistically significantly different (c^2^ test, *P* > 0.05, comparisons per two followed by Bonferoni correction for multiple comparisons).

The differences among populations in the duration of preadult stages were significant as well (Table 2). For example, the difference in egg to adult developmental time between Kenyan and the Guatemalan population was almost three days (Table 2). Immature stage-specific comparisons indicated that medfly populations differed most notably (≍ 2 days) in larval duration and least in egg duration (Table 2).

**Table 2:** Mean duration (days) of immature stages (males and females) of six medfly populations reared on an artificial diet in the laboratory (25°C)

	Mean developmental time (days ± SE)			
	
Population	Egg	Larvae	Pupae	Egg–Adult
Kenya	1.96 ± 0.01 c	5.71 ± 0.02 e	9.20 ± 0.03 d	16.87 ± 0.04 d
Portugal	1.97 ± 0.01 c	6.26 ± 0.06 c	9.92 ± 0.03 b	18.16 ± 0.06 c
Greece	2.01 ± 0.01 b	6.98 ± 0.08 b	9.76 ± 0.03 c	18.76 ± 0.08 b
Hawaii	2.01 ± 0.01 b	5.92 ± 0.04 d	10.4 ± 0.04 a	18.32 ± 0.04 bc
Brazil	2.02 ± 0.01 b	6.13 ± 0.04 c	9.60 ± 0.04 c	17.83 ± 0.06 c
Guatemala	2.25 ± 0.01 a	7.43 ± 0.03 a	10.0 ± 0.03 b	19.68 ± 0.04 a

Averages within the same column followed by the same letter are not statistically significantly different (log-rank test; *P* > 0.05).

The estimation of beta coefficients of the model (1a) shows a significant effect of population on the duration of egg (c^2^ = 371.8, df = 5, *P* < 0.001) (see Appendix S1 in supporting information), larval (*x*^2^ = 354.8, df = 5, *P* < 0.001) (see Appendix S2), pupal (*x*^2^ = 147.7, df = 5, *P* < 0.001) (see Appendix S3), and egg to adult (c^2^ = 528.3, df = 5, *P* < 0.001) (Table 3) developmental periods. The negative values of the beta coefficients (Table 3) denote that an individual originating from Guatemala, Greece, Brazil, Portugal, and Hawaii reached adulthood significantly later than an individual from Kenya (baseline).

**Table 3:** Variables of the Cox proportional hazards model on the effect of population (covariate) on the duration of total immature development (egg–adult) of six medfly populations reared on an artificial diet in the laboratory (25°C). Individuals from Kenya form the baseline

Source of variation	β	SE	Exp(β)	*P*
Populations				<0.001
Portugal	−1.905	0.127	0.149	<0.001
Greece	−2.277	0.130	0.103	<0.001
Hawaii	−1.708	0.124	0.181	<0.001
Brazil	−1.520	0.122	0.219	<0.001
Guatemala	−2.989	0.133	0.050	<0.001

### Population increase parameters

**Figure 2:**
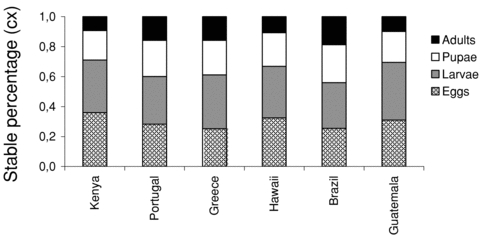
Expected stable age distribution of six medfly populations reared under stable conditions in the laboratory (25°C). Data regarding adult demographic traits were derived from Diamantidis et al. 2009.

Data on the life-history traits of immatures were combined with earlier ones on adult demography of the above six populations (Diamantidis et al. 2009) to obtain population parameters. Our analysis, combining preadult survival and developmental rates, adult female survival, and female fecundity revealed significant differences among medfly populations tested in population growth (r) (Table 4). The population from Kenya scored the highest intrinsic rate of increase compared to the remaining five populations. The highest net reproductive rate was estimated for flies originating from Greece, followed by the Guatemalan, Hawaiian, Kenyan, Brazilian, and Portuguese flies, respectively (Table 4). Mean generation time among all populations tested ranged between approximately 42 days (Kenya) and 63 days (Greece) with the difference being significant only between the above two populations. Overall, population parameters in our study were in general agreement with the ones reported by previous studies (Carey 1982; Vargas et al. 2000). The expected stable age distribution of the six populations tested under stable conditions in the laboratory (25°C) is shown in Figure 2. For all six populations tested, immature stages represent the largest part of the theoretical stable age distribution (Fig. 2). However, considerable variation among populations was observed in the expected proportion of adults. For example, while the expected proportion of adults was ≍ 9% for populations from Kenya and Guatemala, it reached almost 19% for the Brazilian one (Fig. 2).

**Table 4:** Population parameters of six medfly populations reared on an artificial diet in the laboratory (25°C). 95% confidence intervals were obtained by bootstrap. Data regarding adult demographic traits were derived from Diamantidis et al. 2009

	Populations					
	
Parameters	Kenya	Portugal	Greece	Hawaii	Brazil	Guatemala
Intrinsic rate of increase (*r*)	0.122	0.086	0.087	0.110	0.082	0.108
	[0.110, 0.151]	[0.070, 0.107]	[0.074, 0.104]	[0.093, 0.125]	[0.067,0.095]	[0.093, 0.128]
Intrinsic birth rate (*b*)	0.144	0.108	0.091	0.124	0.103	0.113
	[0.121, 0.176]	[0.09, 0.141]	[0.075, 0.110]	[0.107, 0.155]	[0.082, 0.131]	[0.102, 0.140]
Intrinsic death rate (*d*)	0.022	0.022	0.004	0.014	0.020	0.005
	[0.012, 0.033]	[0.014, 0.033]	[0.001, 0.009]	[0.006, 0.022]	[0.010, 0.033]	[0.001, 0.009]
Net reproductive rate (*R*_0_)	164.5	135.6	237.8	176.0	142.0	187.9
	[129.6, 192.7]	[100.8, 170.6]	[192.9, 282.9]	[142.6, 210.3]	[110,8, 173,4]	[151.6, 227.8]
Doubling time (DT)	5.66	8.03	7.94	6.29	8.38	6.40
	[4.58, 6.26]	[6.40, 9.81]	[6.63, 9.28]	[5.49, 7.40]	[7.22, 10.25]	[5.37, 7.41]
Mean generation time (*T*)	41.7	56.8	62.7	46.9	59.9	48.4
	[32.2, 47.3]	[42.6, 72.5]	[50.6, 75.5]	[39.4, 56.9]	[49.3, 75.8]	[39.0, 57.9]
Average age in stable population (ā)	6.5	9.2	10.0	7.5	10.2	7.8
Stable percentage (*cx*)	[5.04, 7.77]	[6.6, 11.2]	[8.06, 12.3]	[5.6, 8.7]	[8.0, 12.7]	[6.0, 8.7]

## Discussion

Our results demonstrate that medfly populations from six global regions exhibit differences in important life-history traits such as preadult survival and duration of immature stages. This result combined with differences in adult survival and reproductive schedule (Diamantidis et al. 2009) account for divergence among populations in key parameters such as the intrinsic rate of population increase. This fact implies that colonizing medfly populations worldwide share different invasion potential.

Medfly populations in our study differed in the duration of immature development with the differences being more pronounced during the larval stage. Kenyan flies reached adulthood sooner than the other five populations tested. Divergent selection in ecologically diverse habitats probably accounts for the observed differences in the duration of immature development among medfly populations. To date, little is known of the selective pressures shaping the life history of immatures in geographically isolated medfly populations. In a Swedish population of the speckled wood butterfly *Paparge aegeria*, the emergence of females in a very limited time window combined with nonoverlapping generations select for shorter immature development of males and protandry, whereas the reverse was observed for a population originating from Madeira, Portugal, probably due to overlapping generations (Gotthard et al. 1994). Since this does not seem to apply to the case of medfly, future research should focus on shedding light on the selective regimes that shape the life history of immatures in different medfly populations. On the other hand, the fact that Kenya represents the source area of this species, as shown by previous genetic studies (Malacrida et al. 1998), may explain why flies from this region complete their preadult development sooner than the other populations tested. Medfly is expected to have the highest diversity of pathogens and specialized predators in this region. Therefore, the evolution of a shorter developmental period, compared to the other populations, may be part of the species strategy to minimize the risk or evade parasitism that operates during larval stage (Papadopoulos and Katsoyannos 2003).

Phenotypic plasticity in our experiment was eliminated by design. Recent studies suggest that medfly exhibits some level of phenotypic plasticity in terms of thermal tolerance (Nyamukondiwa et al. 2010; Nyamukondiwa and Terblanche 2010). However, in these studies the experimental protocols used included medfly acclimation in temperatures that, in many cases, are extreme for medfly survival (−5°C, 41°C). Exposure in such extreme temperature regimes is indeed expected to induce high levels of phenotypic plasticity. However, in our study all six medfly populations were maintained under stable temperature (25 ± 1°C) that is considered very close to optimal growth conditions, not only for medfly, but for other tephritid species as well. Therefore, medfly populations under such optimal conditions are expected to exhibit low levels of phenotypic plasticity compared to extreme environmental regimes. In addition, the genetic differentiation of geographically isolated medfly populations has been demonstrated in a series of previous studies (Malacrida et al. 2007 and references therein). This genetic differentiation provides strong indication that differences among medfly populations worldwide in life-history traits, as observed in our study, represent evolutionary responses. Furthermore, by using the same common garden experimental approach (identical conditions for all medfly populations) as in the current study, we have demonstrated that medfly populations worldwide exhibit significant differences in important life-history traits such as: (a) sexual signaling (Diamantidis et al. 2008b), (b) adult survival and reproduction patterns (Diamantidis et al. 2009), and (c) domestication in the laboratory (Diamantidis et al. 2011). Therefore, we believe that the observed differences among medfly populations in our study represent evolutionary responses as a result of adaptation to ecologically diverse habitats.

Biological invasions are spatially and temporally dynamic processes that can be divided into three distinct phases: (a) arrival, (b) establishment, and (c) spread (Liebhold and Tobin 2008). During the arrival phase, medfly life-history traits, such as larval developmental duration and mortality may affect propagule size and therefore the outcome of invasion. During the critical period of establishment, invading medfly populations are affected by both demographic (Engen et al. 1998; Sax and Brown 2000) and environmental stochasticity that may ultimately lead small founder populations to extinction due to negative growth rates (Simberloff 1988). These negative effects may be intensified when combined with the “Allee effect,” which is the minimum density of a population that assures its persistence in a specific area. The “Allee effect” is directly connected with propagule pressure and its role during the establishment phase of biological invasions is receiving increased attention (Taylor and Hastings 2005). It is clear that colonizing medfly populations worldwide face the probability of extinction during their establishment into new regions. Despite the fact that establishment may require a different combination of life-history traits depending on the nature of the invaded habitat (e.g., human-disturbed vs. less altered) (Delatte et al. 2009), and traits necessary for establishment may vary across invading insect species (Sakai et al. 2001), it is widely recognized that rapid demographic growth promotes the persistence of an invading population in a novel environment (Kneitel and Chase 2004). For example, Crawley et al. (1986) found that insect species with the highest intrinsic growth rates, introduced as biological control agents, exhibited a higher probability of successful establishment. Therefore, the differential demographic growth potential of different medfly populations, as observed in our study, may strongly affect their overall invasion dynamics by affecting the probability of extinction during the critical establishment phase of biological invasions.

Different growth rates among invading populations may also affect their spread during the last phase of an invasion. Invasive populations may follow different modes of spread. For example, in the case of continuous spread, a colonizing population moves along its front often following a simple diffusion model, with the intrinsic rate of population increase representing a critical factor regulating the rate of continuous range expansion into neighboring regions (Andow et al. 1990). As a consequence, medfly populations with different growth rates may also differ in their ability to spread into previously unoccupied areas. On the other hand, spread of invading insect populations may follow a more complicated pattern of spread, with individuals being transferred over long-range distances mediated mainly by anthropogenic activities. This results in the formation of isolated colonies that ultimately unite with the main population front (Liebhold and Tobin 2008). In this case population growth rates may play a crucial role in the rate of dispersal, since both isolated colonies formed by long-range dispersal and populations standing on the boundaries of the population front are generally of low density and therefore subjected to stochasticity and Allee effects as in the establishment phase of biological invasions (Keitt et al. 2001; Taylor and Hastings 2005). Rapid demographic growth may promote the rate of spread by counteracting such negative effects, while populations with low growth rates may face extinction resulting to a decelerating speed of invasion since the spread phase may have to “start from scratch” (Sakai et al. 2001). Hence, invading medfly populations with differential growth rates may also exhibit significant variation in invasion dynamics due to differences in spread rates.

However, it should be borne in mind that a high intrinsic rate of increase alone cannot define the outcome of invasion (Lawton et al. 1986). In an elegant study involving a comparison of life-history traits among endemic and successive invading fruit fly species in La Reunion Island, Duyck et al. (2007) concluded that traits crucial for the success of invasion were the ones that favored competition rather than colonization. Another factor widely recognized as important, especially during the spread phase of invasion, is habitat variability. By thoroughly analyzing medfly capture data in California, Carey (1996) concluded that mountain topography, valleys, rivers, and shorelines may channel dispersal of an established population through paths of least resistance. Therefore, successful invasion depends not only on the life history of the invader, but also on climatic, geographical, biological, and community barriers such as biodiversity and ecosystems resistance (Harmon et al. 2009).

Our results show that the population from Kenya exhibits the highest growth rate among the other populations tested, as indicated by its intrinsic rate of increase under stable conditions in the laboratory. Taking into consideration the loss of genetic variability, the establishment of an insect colony to the laboratory bears resemblance to the early phases of an invasion event. In the latter case, a significant loss of genetic variability may arise as the result of a limited number of invading individuals (founders), and the small population size in the following few generations (Dlugosch and Parker 2008). On the other hand, bottleneck effects that operate during the initial stages of artificial rearing in the laboratory may drastically decrease the genetic variability of the population (Miyatake 1998; Hoffmann et al. 2001). Adaptation to a novel environment may be accomplished with very low genetic variability (Zayed et al. 2007). However, it is widely recognized that the adaptive ability of a population to a novel environment is promoted by high within-population genetic variability, which is related to both presence of multiple alleles and different allele combinations per locus (Ciosi et al. 2008). Mean expected heterozygosity represents also an important factor during adaptation processes since it affects the dynamics of individuals to produce descendants with high genetic variability (Futuyma 2005). There is mounting evidence that populations derived from Kenya are the most highly polymorphic in both terms of mean number of alleles per locus and mean expected heterozygosity (Malacrida et al. 1998; Bonizzoni et al. 2000; Gasperi et al. 2002). Therefore, it seems plausible that the highest growth rate of the ancestral Kenyan population in stable laboratory conditions stems from its sufficient genetic load that provides flexibility for better adaptation to captivity. However, this fact does not necessarily confers to the Kenyan population an invasiveness advantage over the other five populations, since the laboratory represents just a single environment that is slightly representative of the whole gamut of ecosystems that could be potentially invaded by medfly.

In conclusion, our results clearly demonstrate that, apart from divergence in adult survival and reproductive patterns (Diamantidis et al. 2009), medfly populations worldwide exhibit also significant differences in preadult survival, developmental rates of immatures and important populations parameters such as the intrinsic rate of increase. This fact may affect invasion dynamics of invading medfly populations, since population growth rates may influence basic population processes that operate mostly during the last two stages of an invasion event, such as establishment and spread. However, it should be noted that our results represent adaptation under standardized conditions in the laboratory. Thus, it would be of great interest to test the demographic response of geographically isolated medfly populations under extreme or marginal conditions to determine if such differences in their invasive potential still exist. Such information may be valuable in designing population suppression measures and managing invasiveness of invading medfly populations worldwide.

## Supporting Information

**Table S1.** Variables of the Cox proportional hazards modelon the effect of population (covariate) on the duration of theembryonic (egg) stage of six medfly populations reared on anartificial diet in the laboratory (25°C). Eggs from Kenya formthe baseline.

**Table S2.** Variables of the Cox proportional hazards modelon the effect of population (covariate) on the duration of larvalstage of six medfly populations reared on an artificial diet in thelaboratory (25°C). Larvae from Kenya form the baseline.

**Table S3.** Variables of the Cox proportional hazards modelon the effect of population (covariate) on the duration of pupalstage of six medfly populations reared on an artificial diet in thelaboratory (25°C). Pupae from Kenya form the baseline.
