# Supplementary material for: Population-specific demography and invasion potential in medfly
Source: Ecol Evol. 2011 Dec;1(4):479–88. doi: 10.1002/ece3.33 (PMC3287340; doi:10.1002/ece3.33)
Supplement: Supplementary file 1 [file ece30001-0479-SD1.doc]

**Table S1.** Variables of the Cox proportional hazards model on the effect of population (covariate) on the duration of the embryonic (egg) stage of six medfly populations reared on an artificial diet in the laboratory (25 ºC). Eggs from Kenya form the baseline.

| Source of variation | *β* | SE | Exp(*β*) | *P* |
| --- | --- | --- | --- | --- |
| Population |  |  |  | < 0.001 |
| Portugal | -0.049 | 0.118 | 0.952 | 0.677 |
| Greece | -0.464 | 0.116 | 0.629 | < 0.001 |
| Hawaii | -0.525 | 0.119 | 0.591 | < 0.001 |
| Brazil | -0.896 | 0.122 | 0.408 | < 0.001 |
| Guatemala | -2.416 | 0.140 | 0.089 | < 0.001 |

**Table S2.** Variables of the Cox proportional hazards model on the effect of population (covariate) on the duration of larval stage of six medfly populations reared on an artificial diet in the laboratory (25 ºC). Larvae from Kenya form the baseline.

| Source of variation | *β* | SE | Exp(*β*) | *P* |
| --- | --- | --- | --- | --- |
| Population |  |  |  | < 0.001 |
| Portugal | -1.154 | 0.124 | 0.315 | < 0.001 |
| Greece | -1.702 | 0.127 | 0.182 | < 0.001 |
| Hawaii | -0.354 | 0.118 | 0.702 | 0.003 |
| Brazil | -0.614 | 0.117 | 0.541 | < 0.001 |
| Guatemala | -2.061 | 0.129 | 0.127 | < 0.001 |

**Table S3.** Variables of the Cox proportional hazards model on the effect of population (covariate) on the duration of pupal stage of six medfly populations reared on an artificial diet in the laboratory (25 ºC). Pupae from Kenya form the baseline.

| Source of variation | *β* | SE | Exp(*β*) | *P* |
| --- | --- | --- | --- | --- |
| Populations |  |  |  | < 0.001 |
| Portugal | -0.997 | 0.120 | 0.369 | < 0.001 |
| Greece | -0.836 | 0.116 | 0.433 | < 0.001 |
| Hawaii | -1.431 | 0.125 | 0.239 | < 0.001 |
| Brazil | -0.736 | 0.116 | 0.479 | < 0.001 |
| Guatemala | -1.070 | 0.117 | 0.343 | < 0.001 |
